# Latest Developments in Insect Sex Pheromone Research and Its Application in Agricultural Pest Management

**DOI:** 10.3390/insects12060484

**Published:** 2021-05-23

**Authors:** Syed Arif Hussain Rizvi, Justin George, Gadi V. P. Reddy, Xinnian Zeng, Angel Guerrero

**Affiliations:** 1 National Agricultural Research Center (NARC), Islamabad 44000, Pakistan; arifrizvi@aup.edu.pk; 2 Southern Insect Management Research Unit, USDA-ARS, Stoneville, MS 38776, USA; justin.george@usda.gov (J.G.); Gadi.Reddy@usda.gov (G.V.P.R.); 3 College of Plant Protection, South China Agricultural University, Guangzhou 510642, China; 4 Department of Biological Chemistry, Institute of Advanced Chemistry of Catalonia-CSIC, 08034 Barcelona, Spain

**Keywords:** sex pheromones, integrated pest management, biosynthesis, pheromone perception, resistance, review

## Abstract

**Simple Summary:**

Insect pheromones are specific natural compounds that meet modern pest control requirements, i.e., species-specificity, lack of toxicity to mammals, environmentally benign, and a component for the Integrated Pest Management of agricultural pests. Therefore, the practical application of insect pheromones, particularly sex pheromones, have had a tremendous success in controlling low density pest populations, and long-term reduction in pest populations with minimal impact on their natural enemies. Mass trapping and mating disruption strategies using sex pheromones have significantly reduced the use of conventional insecticides, thereby providing sustainable and ecofriendly pest management in agricultural crops. In this review, we summarize the latest developments in sex pheromone research, mechanisms of sex pheromone perception, and its practical application in agricultural pest management.

**Abstract:**

Since the first identification of the silkworm moth sex pheromone in 1959, significant research has been reported on identifying and unravelling the sex pheromone mechanisms of hundreds of insect species. In the past two decades, the number of research studies on new insect pheromones, pheromone biosynthesis, mode of action, peripheral olfactory and neural mechanisms, and their practical applications in Integrated Pest Management has increased dramatically. An interdisciplinary approach that uses the advances and new techniques in analytical chemistry, chemical ecology, neurophysiology, genetics, and evolutionary and molecular biology has helped us to better understand the pheromone perception mechanisms and its practical application in agricultural pest management. In this review, we present the most recent developments in pheromone research and its application in the past two decades.

## 1. Introduction

**Figure 1:**
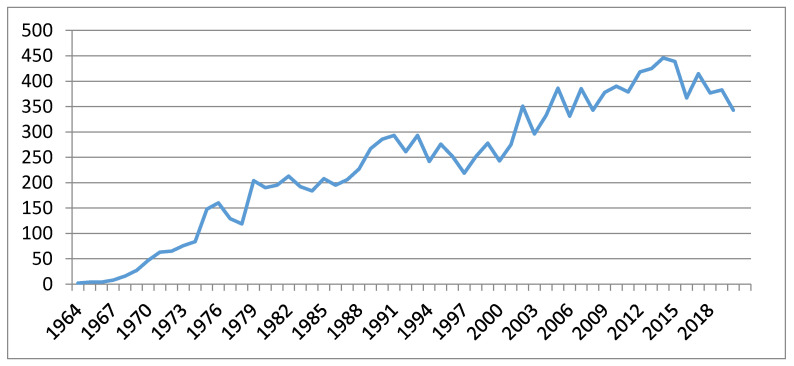
Number of publications on insect sex pheromones appeared in the literature (1964–2020).

Sex pheromones are chemical signals emitted by an organism that elicit a sexual response in a member of the opposite sex of the same species [1,2]. Since the structural characterization of the first sex pheromone of the silkworm moth *Bombyx mori* in 1959 [3,4], more than 600 species [5] of lepidopteran pheromones have been identified. Their main features, e.g., species-specificity, non-toxicity to mammals and other beneficial organisms, their activity in minute amounts, and rapid degradation in the environment were soon envisioned to be promising tools for controlling insect pests, estimating pest populations, detecting the entry and progress of invasive pests, and preserving endangered species [6,7,8]. In fact, in recent years the most successful practical applications of sex pheromones in integrated pest management (IPM) have been the monitoring of pest populations, mass trapping, mating disruption, and push-pull strategies [7,9,10,11]. The number of articles appearing in the literature on these subjects has dramatically increased over the years, particularly in the past two decades (Figure 1), with the sex pheromones of the genera *Helicoverpa*, *Spodoptera*, *Grapholita* and *Cydia* most frequently cited (Table 1). In this review, a literature search, including patents, was conducted by the following search tools and databases: Web of Science, CABI, Agricola, SciFinder, Google Scholar, The Pherobase, and Espacenet. The following keywords were chosen: sex pheromone, sex pheromone autodetection, sex pheromone perception mechanism, sex pheromone biosynthesis, resistance to pheromone, pheromone and biological control agents, the application of pheromones in IPM, and push-pull strategy.

Sex pheromones are mainly produced by females and used as attractant compounds to show the presence of potential mating partners and their reproductive status [8,11]. Sex pheromones comprise sex attractant pheromones, which induce upwind oriented movements to the conspecific individual, and courtship pheromones, which elicit a variety of close-range responses in the insect partner [12,13]. Since the first pheromone discovery, the rapid progress of methodologies developed to identify new pheromones, mainly GC, GC-MS, NMR, electrophysiological techniques [electroantennography (EAG), gas chromatography coupled to electroantennography (GC-EAD), single sensillum recordings (SSR), and coupled GC-SSR], have allowed the identification of thousands of compounds as insect sex pheromones [14] (Table 2 contains new sex pheromones and sex pheromone components recently identified from insect pests in the period 2010–2020 and the corresponding references [15,16,17,18,19,20,21,22,23,24,25,26,27,28,29,30,31,32]). In addition, an interdisciplinary approach involving advances in analytical chemistry, neurophysiology, genetics, and molecular biology have improved our understanding of insect chemical communication and behavior to the level of discrete neural circuits [8].

Insects’ sex pheromones are generally blends of two or more compounds and only in few cases, one chemical, usually the primary component, is efficient to attract conspecifics and mate [33,34]. Löfstedt et al. [35] have reported different types of moth pheromones based on their production, chemical structure, and biosynthetic origin. Type I pheromones are C_10_–C_18_ monounsaturated or diunsaturated acetates, alcohols, and aldehydes [36] and constitute ca. 75% of all known moth sex pheromone components. Type II pheromones are polyunsaturated straight-chain hydrocarbons and the corresponding epoxide derivatives with C_17_–C_25_ carbon atoms [36] and comprise about 15% of the moth pheromones reported. In addition to Types I and II, Löfstedt et al. [35] proposed to extent this classification by defining two more pheromone Types 0 and III. Type III pheromones include saturated and unsaturated hydrocarbons, which may be functionalized, containing one or more methyl branches. Type 0 pheromones, in turn, consist of short chain methylcarbinols and methylketones and have been found more recently in Eriocraniidae and caddisflies (Trichoptera). Another group of pheromones comprising propionate esters of secondary alcohols, methyl-branched secondary alcohols, methyl-branched methylketones, and straight-chain (*Z*)-7-alken-11-ones cannot be categorized in any of the Types 0-III pheromone groups because their structures are not clearly biosynthetically related to any of those classifications [35].

The chemical structure of pheromones is widely diverse. Thus, many of them are hydrocarbons, alcohols, esters, epoxides, aldehydes, ketones, lactones, carboxylic acids, isoprenoids, and triacylglycerides [8,37]. This structural diversity is the key for the pheromone specificity but, in a number of species in Lepidoptera, an appropriate combination of the pheromone components in specific ratio renders the pheromone species-specific. In this review, we present an update of the latest developments of insect sex pheromones reported as useful, environmentally benign, tools for the management of agricultural pests in IPM programs including the mechanisms of pheromone perception, interactions with biological control agents, autodetection, and resistance. An excellent book covering the pheromone communication in moths up to 2015 has recently appeared in the literature [38].

## 2. Sex Pheromone Biosynthesis

Sex pheromone components are C_10_–C_18_ straight chain unsaturated compounds with an oxygenated functional group [39]. In many Lepidoptera species, sex pheromone production is regulated by the pheromone biosynthesis-activating neuropeptide (PBAN), a neurohormone originated in the subesophageal ganglion and released into the hemolymph to operate directly on the pheromone gland (PG) [40], which in turn activates functional group modification enzymes [41] or acetyl-coenzyme A carboxylase (ACC) [42]. In the first step of pheromone biosynthesis, the carboxylation of acetyl-CoA to malonyl-CoA is catalyzed by ACC, followed by fatty acid synthase action which leads to production of saturated fatty acids (C18:0 and C16:0) [42].

Through a series of enzymatic reactions, i.e., desaturation, chain-shortening reaction, reduction, acetylation, and oxidation, the palmitic or stearic acids are then converted to the final pheromone components in a stepwise manner [41,43]. Therefore, different enzymes are likely to be involved in different reactions, and to date, the genes encoding the essential enzymes—fatty acid desaturases (FADs), fatty acid reductases (FARs), chain shortening via peroxisomal β-oxidation, and chain elongation—have been functionally identified. The specificity of the enzymes and their combinations allow formation of an immense array of pheromone components with different chain lengths, unsaturations, and functional groups [5,35]. The discovery of the biosynthetic enzymes is generally accomplished by comparative analysis of gene expression in the female PGs relative to control tissues. The genes that are overexpressed in the pheromone glands are candidate genes to be involved in pheromone biosynthesis, such as desaturases, reductases, etc. The enzymes are expressed in heterologous hosts, such as the yeast *Saccharomyces cerevisiae* [44], insect cells [45], or plants [46], and functionally assayed. Transcriptome data from pheromone glands have been reported for many Lepidopterans, such as *Agrotis segetum*, *Ephestia cautella*, *Pectinophora gossypiella*, *Plutella xylostella*, *Spodoptera exigua*, and *Spodoptera litura* [5]. Transcriptomes from many other insects can be found in the database http://www.insect-genome.com (accessed on 8 March 2021) [47].

FADs are the most intensively reported class of enzymes involved in moth sex pheromone biosynthesis and, thus, more than 50 FADs have been functionally characterized [48]. They are able to display different specificities and introduce double bonds in different positions and geometry. The most common desaturation processes occur in positions Δ9, f.i. in *Mamestra brassicae* [43], *Trichoplusia ni* [49], and *S. litura* [50]; and Δ11, f.i. *Ostrinia* spp. [51], but they can produce also unsaturations at Δ5 in *Ctenopseustis obliquana* and *C. herana* [52], Δ6 in *Antheraea pernyi* [53], Δ8 in *Dendrolimus punctatus* [54], Δ10 in *Planotortrix octo* [55], Δ14 in *Ostrinia furnacalis* [56], and Δ1 in *Operophtera brumata* [57]. A multi-functional Δ10–Δ12 desaturase was found in *B. mori* pheromone biosynthesis [45], Δ11-Δ12 desaturases were noticed in *S. exigua* and *S. litura* [58], and a Δ11–Δ13 desaturase was characterized in *Thaumetopoea pityocampa* [59]. In the case of the homologous Δ5 FAD of *S. litura* (SlitDes5) and *S. exigua* (SexiDes5), the full-length sequences of SexiDes5 (1017 bp) and SlitDes5 (1017 bp), were obtained from the pheromone gland cDNA library transcripts [60], and both encoded proteins with 339 amino acids. In the heterologous expression system, both desaturases inserted a *cis* double bond at Δ11 position in palmitic, myristic and stearic acids. However, while both enzymes introduce only a *cis* double bond in saturated C16 and C18 substrates, in C14 substrates both *cis* and *trans* unsaturations are created. The differences between substrates and the geometry of the substrate-binding tunnel might influence the regioselectivity and stereospecificity of the desaturation reaction [61].

FARs catalyze the reduction of fatty acylCoA precursors into fatty alcohols in a two-step reaction without releasing the intermediate aldehyde forms [62]. They play an essential role in regulating the final steps of sex pheromone biosynthesis in some moths, and have been functionally identified in species from the genera *Agrotis, Bicyclus*, *Bombyx*, *Helicoverpa*, *Heliothis*, *Ostrinia*, *Spodoptera*, and *Yponomeuta* [48]. Reductases from *Helicoverpa* spp. and *Heliothis* spp. can act on a broad range of C8 to C16 fatty acids, preferentially on C14 substrates [63]. In the European and Asian corn borers, *Ostrinia nubilalis* and *O. furnacalis*, in vivo labeling studies demonstrated that the selectivity of the reductase system could modulate ratios among final pheromone components by exclusive conversion of specific acid moieties into their corresponding alcohols [64]. Four reductases from *Spodoptera* spp. showed different selectivity for C14 and C16 fatty acids, whereas SexpgFAR I and SlitpgFAR I selectively act on C16 fatty acids, SexpgFAR II, and SlitpgFAR II preferred C14 fatty acids as substrates [65]. The small ermine moths, namely *Yponomeuta evonymellus*, *Yponomeuta padellus*, and *Yponomeuta rorellus* use pheromone blends made of structurally related C14 and C16 fatty alcohols and their derivatives [66]. In *Y. evonymellus* and *Y. padellus*, the two primary pheromone components are Z11–14:OAc and E11–14:OAc, and complete reproductive isolation is ensured by the use of additional pheromone components. In contrast, *Y. rorellus* uses only the saturated acetate 14:OAc as a pheromone. By screening the PG FAR genes, Liénard and coworkers [66] demonstrated that the reduction step of long-chain C14- and C16-acyl pheromone precursors found in the three insects is accounted for by a single PG-specific FAR.

There are other important enzymes that have not been functionally confirmed but are postulated to be involved in the sex pheromone biosynthesis pathway. For example, biochemical studies have suggested that acetyltransferases (ACTs) and alcohol dehydrogenases (ADHs) play a crucial role by converting fatty alcohols into the corresponding fatty acetates and aldehydes, respectively, since they constitute the last step of the pheromone biosynthetic pathway in many moths. Currently, no insect ACTs have been characterized to esterify fatty alcohols, in contrast to plants [67,68] and yeast [69] from which a number of ACTs (EC: 2.3.1.84) were cloned. In a recent study on the pheromone biosynthetic pathway of *A. segetum*, Ding and Löfstedt [44] expressed 34 genes potentially encoding for ACTs but none of them successfully converted fatty alcohols into the corresponding acetates. The sex pheromone of *P. xylostella* is a mixture of (*Z*)-11-hexadecenal, (*Z*)-11-hexadecenyl acetate, and (*Z*)-11-hexadecenol in 8:100:18 ratio [70]. In its PG transcriptome two transcripts encoding proteins homologous to acetyltransferases ACT1 and ACT2 were found [71]. ACT1 was homologous to acetyl-CoA ACT from *Amyelois transitella* and ACT2 had similar amino acid sequence to the ACT gene from *S. litura*. However, neither of them was homologue to the genes belonging to the group of ACTs EC 2.3.1.84.

With regard to pheromone aldehydes, Chen et al. [71] found that the amino acid sequences encoded by five transcripts in *P. xylostella* PG transcriptome resemble alcohol dehydrogenase (ADH, EC 1.1.1.1) genes, but none of them were cloned and characterized. ADHs are a group of enzymes that facilitate interconversion between alcohols and aldehydes with the reduction of NAD^+^ to NADH in the biosynthetic pathway of pheromone aldehydes. β-Oxidation enzymes are supposedly involved in the biosynthesis of pheromones of shorter chain lengths and may play an important role in regulating the ratio of sex pheromone compounds with different carbon lengths. The genes involved in β-oxidation have been identified in some moth PG tissues [72] but not yet characterized.

## 3. Mechanisms of Insect Sex Pheromone Perception

Insects detect volatile odorants/pheromone molecules using olfactory receptor (OR) sensilla present on their antennae and maxillary palps [73]. Pheromones and other odorant molecules that are absorbed on the cuticular surface of an olfactory sensillum diffuse inside through olfactory pores and the pore tubule [74]. The sensillum lymph contains the pheromone binding proteins (PBPs) that bind with volatile pheromones and solubilize them to pass across the sensillum lymph to activate pheromone receptors [75]. The structure and arrangement of olfactory sensilla and olfactory sensory neurons (OSNs) on the antennae and palps of insects are very specialized and optimized to detect odorants, especially sex pheromones in the case of male antennae. OSNs carry the olfactory information from the periphery to the antennal lobe in the insect brain. Many studies have reported the structure and peripheral olfactory mechanisms of odorant perception in insects [73,76,77]. Other works that have investigated these different olfactory elements, and the cellular and molecular mechanisms of volatile pheromone signal detection in olfactory sensilla have been published in recent review publications [75,78,79]. Recent studies have also reported on the evolution of olfactory circuits and processing of these information in the higher olfactory centers in the insect brain [80,81].

The peripheral olfactory hairs or olfactory sensilla that house the OSNs play an essential role in odorants perception, especially sex pheromones. Different morphological types and distribution of olfactory sensilla have been reported in multiple insect species based on their ecological niches. For example, the long trichoid sensilla house the OSNs tuned to sex pheromones in many moth species, whereas the antennae in flies and wasps contain basiconic and placoid sensilla, respectively, which are the most common peripheral olfactory hairs [82]. In the last decade, significant improvements have been made in understanding the ultrastructural features of peripheral olfactory sensilla and its correlation with neuronal mechanisms using electron microscopy and genetic labelling, which has enabled the integration of morphological and molecular information in different insects, such as *Drosophila* sp. and *B. mori* [83,84]. In this context, a “Cryochem method” has been developed and used in *Drosophila melanogaster*. This method rehydrates cryofixed and high-pressure frozen samples for creating three-dimensional reconstructions of genetically marked OSNs in different sensilla by serial block-face scanning electron microscopy. It is found useful in providing insights into the relationship between OSN anatomy and olfactory physiology [83,84]. A recent review of these new molecular tools and mechanisms of olfactory detection in insects has been published [85].

A significant amount of research has been done in the last decade to understand the odorant receptors (ORs) [86,87] involved in chemo-electric signal transduction and processing of odorant signals in the insect brain. Sato et al. [88] and Wicher et al. [89] reported the role of OR co-receptor (Orco) that is characteristic for each OR-expressing sensory neuron. Orco acts as heteromeric ligand-gated and cyclic-nucleotide-activated cation channel, and is highly conserved across insect species and orders [90]. Sakurai et al. [91] published a detailed review on the neural and molecular mechanisms involved in sex pheromone perception and processing in the silkworm moth, *B. mori*. Rapid progress in the sequencing technologies, genomics, and transcriptomics of insect olfactory tissues has helped identify odorant receptor (OR) gene families for multiple insect species in recent years [79]. These new technologies have helped improve our knowledge of insect sensory systems and their practical application in product development for pest management.

Many studies have been published to understand the role of odorant binding proteins (OBPs), pheromone binding proteins (PBPs), sensory neuron membrane proteins (SNMPs), and sex pheromone receptors that are involved in the transport and central processing of pheromone molecules in *Drosophila* and different moth species [75,92,93]. Genomic and transcriptomic analysis have allowed the identification of new sensory neuron membrane proteins (SNMPs) in Lepidoptera, Diptera, Coleoptera, and Hemiptera (see f.i. the excellent review by Cassau and Krieger [94]), and their role in pheromone signaling has been noticed [95]. Very recently, it has been disclosed that a new sensory membrane protein, HarmSNMP1, plays an essential role in the detection of long-chain sex pheromones in *Helicoverpa armigera,* but this protein was not required for detecting shorter chain sex pheromones of the same species [96]. Zhao et al. [97] reported the identification of 49 OBPs and 5 chemosensory proteins (CSPs) in the chive gnat *Bradysia odoriphaga*, and Zhang et al. [98] identified 64 ORs, 24 OBPs and 19 CSPs after analyzing transcripts expressed in chemosensory organs of the beet armyworm *S. exigua.* Initial studies were reported in *Drosophila*, where the pheromone binding protein LUSH carries the hydrophobic pheromone, *cis*-11-vaccenyl acetate (cVA), through the aqueous sensillum lymph to the olfactory neuron dendrites [99]. In recent years, RNAi techniques have been widely used to identify OBPs and PBPs in insects [100]. Oliveira et al. [101] has reported the use of RNAi technique to identify the PBP RproOBP27, involved in sex pheromone detection of *Rhodnius prolixus.* Identification and molecular characterization of PBPs from multiple insect species, such as *Cydia pomonella* [100], *Chilo suppressalis* [102], *Loxostege sticticalis* [103], and *Conogethes pinicolalis* [104] have been reported in recent years. PBP1 from *S. exigua* [105] and *Cyrtotrachelus buqueti* [106] can also bind to plant volatile compounds, such as benzaldehyde, linalool, indole, and other carboxylic acids, in addition to pheromone molecules. Also, a dual role of the GmolOBP7 from the oriental fruit moth *G. molesta* in the detection of both sex pheromone components and host plant volatiles has been disclosed [107].

An insect’s age and the physiological state also affect its responsiveness to sex pheromones and other host plant volatiles. A very good example is the mating-dependent olfactory plasticity exhibited by *Agrotis ipsilon* males. The male copulates only once in a scotophase and results in a temporary inhibition of attraction to the sex pheromone [108], but in turn has no effect in their response to plant odors. This olfactory plasticity allows mated *A. ipsilon* males to transiently block their central responses to pheromones after mating. This leads to an increased non-pheromonal odor detection allowing more efficient finding of food sources in a natural environment [108]. Kromann et al. [109] studied this olfactory plasticity in *Spodoptera littoralis* and showed that newly mated males stopped responding to pheromones and host odors but not to food odors. In addition, Hatano et al. [110] showed that (*E*)-4,8-dimethyl-1,3,7-nonatriene (DMNT), a key cotton volatile compound used by natural enemies in finding prey, is used by females and males of *S. littoralis* to avoid induced plant sites and calling females, respectively. This chemical suppressed responses to the main pheromone component, (*Z*,*E*)-9,11-tetradecadienyl acetate, and to (*Z*)-3-hexenyl acetate, a host plant attractant. In neurophysiological experiments, the compound interfered with host plant and mate location through suppression of olfactory signaling pathways. Using Ca^2+^ imaging, the authors demonstrated that the major component of the pheromone elicited calcium responses in the cumulus, the largest glomerulus of the MGC, but addition of DMNT suppressed them [110]. As DMNT attracts natural enemies and deters herbivores, it may be useful in the development of push-pull strategies.

Jarriault et al. [111] found that the sensitivity of antennal lobe neurons to pheromones increases with age and is dependent on the level of octopamine and juvenile hormone (JH) in *A. ipsilon*, but posterior studies by these authors indicated that it is not mediated by octopamine or serotonin [112]. Follow-up studies by Deisig et al. [113] revealed that in *A. ipsilon* plant odors, such as heptanal, reduce pheromone sensitivity at the macroglomerular complex (MGC) level, resulting in an improved temporal resolution of pheromone pulses by antennal lobe (AL) output neurons [114]. In addition, heptanal activated the specialist olfactory receptor neuron (ORN) for (Z)-7-dodecenyl acetate, one of the pheromone components, and altered the ratio responses of pheromone-sensitive neurons [115]. In another noctuid moth, *H. armigera*, Ian et al. [116] used calcium imaging to reveal a reduced increase of intracellular calcium levels when stimulated with a blend of sex pheromone and complex plant odors as compared to individual odor application. Recent studies by Borrero-Echeverry et al. [117] reported that host plant volatiles enhance the selectivity for conspecific pheromone blends in the noctuid moth *S. littoralis*, and provided evidence for the evolution of pheromone specificity within a host plant odor environment, although the neural mechanisms are unknown. Other studies have reported that interactions between sex pheromone compounds and some plant-derived signals occur in olfactory receptor neurons (ORNs) [118,119].

Recent studies have shown a significant interest in understanding the neurophysiological mechanisms that regulate the interaction between female sex pheromone and behaviorally active host plant odorants by using functional imaging of the antennal lobe (AL) and intracellular recordings (IRs) of projection neurons (PNs) that transmit olfactory signals to higher centers of insect brain [120]. Galizia et al. [121] reported the specific role of the macroglomerular complex (MGC) in pheromone coding, and how the sexually isomorphic, ordinary glomeruli codes for plant volatile information. The olfactory coding of sex pheromones and general plant odors are supposed to occur in these different pathways of the olfactory system of insects. However, studies by Varela et al. [122] and Trona et al. [123] revealed some interesting observations on the processing of sex pheromones and general odorants by moths in the Tortricid family. Varela et al. reported that in *Grapholita molesta* the processing of sex pheromones occurs in olfactory glomeruli (OG) rather than in the macroglomerular complex (MGC), whereas in *C. pomonella* no clear segregation between the pheromone and the general odor were observed. Both odor classes were represented in the MGC and in OG [123] and were correlated with behavioral responses [120]. In the codling moth *C. pomonella*, it was noticed that the macroglomerular complex (MGC) in the antennal lobe (AL), involved in pheromone perception, showed an enhanced response to blends of pheromone and plant signals, whereas the response in glomeruli surrounding the MGC was suppressed [123]. This effect implied a higher attraction of males to blends of female sex pheromone and plant odor compared with single compounds. These findings show that, in nature, sex pheromone and plant odors are perceived as an ensemble, and mating and habitat cues are coded as blends in the MGC of the AL highlighting the dual role of plant signals in habitat selection and in premating sexual communication [120]. Very recently, reviews on the plasticity and modulation of olfactory circuits of insects in a complex environment with different odorants and pheromones, and new neuroecological studies directed to understand the evolution of insect sensory systems, have been published [124,125]. These studies could eventually provide us with potential tools to protect endangered species and reduce the risk for the invasion of alien species.

Research studies have also reported the effects of sex-pheromone exposure on non-sexual behaviors, such as gustatory perception and habituation (a non-associative learning) in male *A. ipsilon* moths. Hostachy et al. [12] used proboscis extension response (PER) assay to investigate the links between reproduction and gustation in *A. ipsilon* by assessing whether their sex-pheromone can modulate sucrose responsiveness and gustatory habituation. Experiments showed that the conspecific sex-pheromone (blend of Z7-dodecenyl-acetate, Z9-tetradecenyl-acetate, and Z11-hexadecenyl-acetate in 4:1:4 ratio) and the hetero-specific sex pheromone (Z5-decenyl acetate) had time-dependent effects on gustatory habituation of *A. ipsilon* moths. This study showed that the sex pheromones can play modulatory roles in gustatory perception in non-social insects, such as moths, and may affect their behavioral plasticity. Follow up studies by Murmu et al. [126] showed that pheromones facilitated both appetitive and aversive olfactory learning in *A. ipsilon* moths. The exposure to the *A. ipsilon* conspecific sex-pheromone before conditioning enhanced appetitive but not aversive learning, while exposure to a heterospecific sex-pheromone component facilitated aversive but not appetitive learning. These modulatory effects of sex pheromones on insects’ learning and memory may have practical applications in developing specific traps that uses attractants or deterrents for pest control.

## 4. Evolutionary Aspects of Olfactory Receptors

Insects detect odorants through olfactory sensory neurons (OSNs), housed within the olfactory sensilla located in the surface of the antennae and maxillary pals [73] The olfactory capacities of an insect depend essentially on the repertoire of expressed olfactory receptor (OR) genes and the functional properties of OR proteins (OBPs), their sensitivity and their variety of responses. These two large gene families, the OBPs and the ORs, are presumably exclusive to insects but when they first appeared in the insect lineage remains to be determined [127]. The ORs form a large and highly divergent gene family, which shows no homology to the OR families of vertebrates. In contrast to the ORs of vertebrates, insect ORs form heteromeric complexes that are typically composed of a single ligand OR and the OR corecepotor ORCO [128]. Many OR repertoires have been identified and unique lineage-specific expansions of OR clades have been observed in different insect orders. This suggests that in each order ORs have followed different evolutionary trajectories as insects have adapted to new ecological niches [127]. This adaptation has been studied through the ORs repertoires from the Diptera *D. melanogaster* [129] and the malaria vector mosquito *Anopheles gambiae* [86], which have demonstrated that the ORs repertoires have been specialized in the detection of ecologically relevant natural products. Apart from Diptera, no other OR repertoire has been functionally characterized. However, De Fouchier et al. [127] have reported a functional analysis of a large array of ORs from the cotton leafworm *S. littoralis*, from which the antennal transcriptome had been previously sequenced [130]. A total of 35 candidate *S. littoralis* receptors (SlitORs) were expressed in *Drosophila* OSNs and identified SlitORs tuned to a variety of odorant molecules, which had been previously shown to be physiologically or behaviorally active in this species [127]. These include host plant and herbivore-induced volatiles, oviposition cues, larval frass volatiles, and pheromone components. The authors reported that receptors have now been deorphanized in 13 different clades of the lepidopteran OR phylogeny, including previous results obtained from *B. mori*, *Epiphyas postvittana*, and *S. exigua*, among other moths. A number of deorphanized receptors had aromatic compounds as their best ligand whereas others were best activated by terpenes and in a lower extent by aliphatic chemicals. Overall and from an evolutionary perspective, the results suggest that receptors to aromatics emerged first and have been more conserved during the evolution of Lepidoptera, whereas receptors to terpenes and the aliphatic compounds emerged more recently and evolved faster (especially aliphatic receptors, which include pheromone receptors) [127]. These properties correlate well with the ecological needs of herbivorous and nectar-feeding insects, such as moths, since aromatics and monoterpenes are the major constituents of plant odors emitted by flowers and leaves. Sex pheromone receptors and a large part of ORs tuned to terpenes and short chain acetates appear to belong to later lineages with a higher rate of evolution.

## 5. Sex Pheromone Autodetection

The phenomenon of a sex pheromone producer insect capable of detecting its conspecific sex pheromone components is termed autodetection [131]. One of the first cases recorded on autodetection was reported by Schneider et al. [132] in which electroantennographic responses to both pheromone components released by females of *Panaxia quadripunctaria* Poda (Lepidoptera, Arctiidae) were equally detected and with similar amplitude by both sexes. It revealed that not only males could detect the female-produced pheromones, but females were not ‘anosmic’ (unable to detect their conspecific sex pheromone) for their own attractant. In some cases, the pheromone only attracts males, thus acting as a sex pheromone, but may induce other behavioral effects on females, e.g., a repellent effect, an advance or delay in calling initiation, or an increase in calling frequency, among others [133]. Sex pheromones are secreted by one sex and cause an intraspecific attractant response and mating in individuals of the opposite sex, but in some rare cases, the pheromone attracts both sexes, functioning more like an aggregation pheromone. The aggregation pheromones are emitted by insects of one sex and cause individuals of both sexes to join for feeding and reproduction. In sex pheromone autodetection, female aggregation may increase the possibilities of mating success or induce dispersal at high population levels.

Until 2015, most of the autodetection studies were focused on Lepidoptera and Coleoptera, of which 28 cases (67%) involved species in the order Lepidoptera, 12 (29%) in the Coleoptera, one (2%) in the Blattodea, and one (2%) in the Diptera [133]. Among Lepidoptera, responses of 7 families (Tortricidae, Noctuidae, Arctiidae, Cossidae, Sesiidae, Yponomeuta, and Pyralidae) out 11 families were detected positive to their sex pheromone by Electroantennography (EAG) and Single Sensillum Recording (SSR) of female antennae, in at least one species within a given family, and only the Saturniidae, Bombycidae, and Geometridae lepidopteran families showed no response to their pheromones.

Bakthavatsalam and coworkers [134] also proved that gravid females of *H. armigera* (Hubner) respond to their pheromone blend (mixture of (*Z*)-11-hexadecenal and (*Z*)-9-hexadecenal in 97:3 ratio) eliciting stronger responses than unmated males. However, virgin or gravid females showed poor response in wind-tunnel studies, and an oviposition bioassay where gravid females were allowed to oviposit in the presence and absence of pheromone odors indicated that there was no difference in the number of eggs laid. Although morphological differences in antennal size and complexity might correlate with differential pheromone detection ability between sexes in some families, such as Saturniidae, many other families such as Tortricidae and Noctuidae were not morphologically different [117]. Moreover, comparing studies of antennae morphology and pheromone sensitivity among various types of sensilla confirmed that sex pheromone detection is not directly related with the gross morphology of antennae [135]. The proteins of PBPs and PRs are crucial in pheromone detection. At least one species in each of the 9 Lepidoptera and Coleoptera families tested contained these proteins (or precursors) [133]. In comparison to males, female antennae exhibited dramatically fewer PBPs and PRs.

Contradictory results were reported in the autodetection of *G. molesta* females. Kuhns et al. [136] found lower mating success after exposure to pheromone but not in calling behavior, while Stelinski et al. [137] reported no effect of pheromone pre-exposure on mating success. Also, Stelinski and coworkers [138] previously reported that females elicited significantly higher EAG responses than solvent-treated controls as well as an advance of the onset of female calling. These contradictory results from two different studies on the same insect highlight variations in behavioral responses, depending possibly on the assay conditions.

As the first report of an insect of the family Gelechiidae exhibiting autodetection, the tomato leafminer *Tuta absoluta* Meyrick (Lepidoptera: Gelechiidae) females exhibited electrophysiological responses to their own pheromone. However, the elicited EAG responses were much lower than those induced by males [139]. The depolarizations displayed by virgin females when stimulated with the binary mixture were significantly higher than those displayed by mated females, although detection of the individual pheromone compounds was generally higher in mated females but not significantly.

A new example of autodetection was found recently in female *Contarinia nasturtii* (Diptera: Cecidomyiidae) 140]. It represents a second family of Diptera exhibiting a case of pheromone autodetection. In both laboratory and field experiments, females exposed to stereospecific and racemic three-component pheromone blends called more frequently and for longer periods than midges in control treatments. Additionally, pre-exposure to stereospecific and racemic pheromone component blends reduced subsequent matings on females (42% and 35%, respectively) vs. 68% of female midges mated under control conditions. The authors concluded that in pheromone-treated fields while more frequent callings may increase the probability of females being detected by males, a reduction in females predisposition to mate would enhance the efficacy of mating disruption experiments [140].

Although the combined ecological and molecular data available suggested that sex pheromones had been detected only in adult moths, Poivet et al. [141] provided a strong evidence that caterpillars can detect and be attracted by the adult sex pheromone of *S. littoralis*. The authors showed that larvae walk towards a sex pheromone source, since their antennae house olfactory receptor neurons (ORNs) that respond to the pheromone and express the PBPs already identified in adults. Moreover, the larvae significantly preferred a food source with the major pheromone component to other lacking the chemical. This unexpected larval behavior may open new expectations in pest control strategies [141]. The responses of olfactory sensilla on the larval antennae of *Heliothis virescens* to specific sex pheromone components were also later reported by single sensillum recordings (SSRs) [142]. Two pheromone receptors HR6 and HR13 were found to be expressed in two and three candidate pheromone response cells, respectively, and other cells expressed PBP1 and PBP2. The results suggested that the responsiveness of larval sensilla to the female sex pheromone is based on similar molecular machinery as in adult male antennae.

As noticed above, the behaviors of autodetection are diverse, and some are even contradictory, such as repelling/attraction or dispersal/aggregation. This suggests that a complete understanding of the autodetection behavior of a species is necessary before applying sex pheromone traps in monitoring insect pest populations and mating disruption strategies.

## 6. Resistance of Insects to Sex Pheromones

To date, there are only a few reports on the development of resistance of insects to sex pheromones. The first report on the potential for evolution of resistance to pheromones was described for the pink bollworm moth, *P. gossypiella* (Saunders) (Lepidoptera: Gelechiidae) by Haynes and coworkers [143]. After an extensive examination of the release rates and blend ratios of pheromonal components emitted by field-collected *P. gossypiella* females, the authors found no evidence of resistance to pheromones applied to cotton fields to disrupt mating. Haynes et al. [143] theorized that while resistance to the *P. gosspiella* pheromone is still an opportunity when used profoundly in managing insect pests as a mating disruptant, there are modern alternative IPM options to prevent the development of resistance to sex pheromones. On the other hand, since mating disruption using synthetic pheromones did not cause any insect mortality, resistance was not likely to emerge. Haynes and Baker [144] stated that a slight change in pheromone emission in insects would represent effective resistance to disruptant pheromones. They found that female *P. gossypiella* from the desert cotton-growing areas of southern California emitted a significantly higher pheromone (20%) in 1984 and 1985 than in 1982 and 1983. They hypothesized that this upsurge could result from selection pressure provided by the continuous application of mating disruptants for population control. However, for blend quality [145] and mating disruption (MD) [146], higher quantity of pheromones have been used than the previous studies.

Evenden and Haynes [147] suggested that MD experiments using the same pheromone blend along the years might affect certain pheromone phenotypes, and therefore modify the chemical communication channels of the insect. This could lead to generation of resistance to MD. In contrast, other authors reported that resistance to MD may be a function of a genetically-based change in the output and reaction to pheromone components of the target pest [148,149].

The first example of resistance to MD was documented on one of Japan’s major tea pests, the smaller tea leafroller moth, *Adoxophyes honmai* Yasuda (Lepidoptera: Tortricidae) [150]. The authors noticed that using (*Z*)-11-tetradecenyl acetate as mating disruptant for four years induced a disruption percentage of pheromone trap catches of 96%, but 14–16 years later the percentage of catches became less than 50%. Moreover, the application of the compound to other previously untreated tea fields induced a disruption higher than 99%. These results brought the authors to term “resistance” of the leafroller to (*Z*)-11-tetradecenyl acetate. In new experiments, application of the 4-component pheromone blend of the insect (63:31:4:2 mixture of (*Z*)-9-tetradecenyl acetate, (*Z*)-11-tetradecenyl acetate, (*E*)-11-tetradecenyl acetate, and 10-methyldodecyl acetate) to disrupt the resistant population induced a 99% disruption and a decrease in larval development [150]. Moreover, resistant males supposedly utilize the widespread odor of (*Z*)-11-tetradecenyl acetate released from MD devices installed in the tea canopy for orientation, while they trace a directional plume of (Z)-9-tetradecenyl acetate emitted from the lure [151].

Tabata et al. [152] noticed that resistant males were attracted to lures with significantly deviated ratios of the pheromone components: 72% responded to the mixture lacking (*Z*)-11-tetradecenyl acetate, which is indispensable for the response behavior of the susceptible males. To avoid this resistance, the authors developed a new strain of resistant insects by rearing field-collected resistant individuals with the pheromone for more than 70 generations [153]. The new males could respond and copulate with their conspecifics even in the presence of 1 mg L^−1^ of the disruptant. In addition, the composition of the sex pheromone blend produced and emitted by females was not changed compared with those of females sensitive to MD.

The continuous exposure or pre-exposure to high concentrations of sex pheromone as in mating disruption experiments can elicit habituation or desensitization [154]. This mechanism implies a reduction of response when pest species are treated with species-specific dispensers [155]. Adaptation of the peripheral neurons system to the sex pheromone has been shown to occur in most moths, but not in all cases where it has been examined, including *G. molesta* [154], *C. pomonella* [156], or *E. postvittana* [157]. In an interesting work, the possible different effects of behavioral interference with high pheromone loadings on four orders of pest insects were studied [157]. The tests were implemented on the light brown apple moth, *E. postvittana* (Lepidoptera: Tortricidae), the citrophilous mealybug, *Pseudococcus calceolariae* (Homoptera: Pseudococcidae), the apple leaf curling midge, *Dasineura mali* (Diptera: Cecidomyiidae), and the Argentine ant, *Linepithema humile* (Hymenoptera: Formicidae). Only in the tortricid moth, pre-exposure of male moths to the main sex pheromone, (*E*)-11-tetradecenyl acetate, significantly reduced their subsequent behavioral responses to the pheromone stimulus compared with the untreated insects, like in usual habituation experiments on pheromone pre-exposure [158]. The insects in the three other orders showed no evidence for habituation to behaviorally active amounts of synthetic pheromone. The authors concluded that the range of mechanisms opened to use in pest management for the three order insects is potentially reduced, and at the same time it raises questions about the adaptive benefit of habituation in Lepidoptera [157].

## 7. Application of Insect Sex Pheromones

Many of the conventional methods using hazardous chemicals for insect pest management have been banned because of their adverse effect on the environment and human health. To overcome the negative effect of synthetic chemicals, many researchers have emphasized the need to develop and formulate eco-friendly and more specific agricultural practices for IPM [159]. The specific characteristics of insect pheromones determine their potential uses as environmental-friendly behavioral regulators in agriculture. On one hand, sex pheromones are species specific compounds with a potential use in population monitoring and mass trapping; on the other hand, pheromones are chemical messengers between the male and female adults of an insect species, with which one sex communicates their sex partners the predisposition and willingness to mating, pointing to a potential use in mating disruption. As early as in the 1970–80s, research studies were undertaken to confirm the possible practical application of pheromones in pest populations monitoring, mass trapping, mating disruption and push-pull strategies [7,9,10,11].

### 7.1. Interactions between Pheromones and Insects Biological Control Agents

The review article by Sharma et al. [160] has covered the most recent literature on the interactions between insect biological control agents and semiochemicals (predominantly pheromones). Therefore, we will discuss the work done in the last two years.

#### 7.1.1. Pheromones vs Entomopathogenic Fungi

It is believed that entomopathogenic fungi act slowly and take time to cause mortality in insects. On the other hand, the combined use of entomopathogenic fungi with chemical insecticides [161] or other entomopathogenic fungi [162] improves microbial control agents’ efficacy. In the same approach, entomopathogenic fungi and pheromones can be used to increase the effectiveness (additive or synergistic) against the target insects. Thus, the simultaneous application of *Metarhizium anisopliae* var. acridum and the aggregation pheromone phenylacetonitrile to *Schistocerca gregaria* Forsskål (Orthoptera: Acrididae) fifth-instar nymphs exhibited an additive interaction [163]. Synergistic interaction was also demonstrated when nymphs were exposed to pheromone first and then treated with *M. anisopliae* var. *acridum*. Thus, Gutiérrez-Cárdenas et al. [164] disclosed the potential of entomopathogenic fungi for the management of adults of *Spodoptera frugiperda* using the auto-dissemination technique. Three isolates of *Beauveria bassiana* (Balsamo-Crivelli) Vuillemin and six isolates of *Metarhizium anisopliae* (Metschnikoff) (Hypocreales: Clavicipitaceae) were reported to be pathogenic to the insect, and the isolate *M. anisopliae* Ma-San Rafel-2 was used for auto-dissemination. The fungus and the synthetic sex pheromone attracted, infected, and killed males 5–8 days after dissemination, paving the way for the potential management of *S. frugiperda* [164]. In the same way, Akutse et al. [165] evaluated the effect of 22 entomopathogenic fungi isolates on the fall armyworm *S. frugiperda*, and their compatibility with the pheromone FALLTRACK lure. All fungal isolates screened were pathogenic to the moths, particularly *Beauveria bassiana* ICIPE 621 and *M. anisopliae* ICIPE 7, which caused 100% mortality of the moths. Both isolates were also found compatible with FALLTRACT lure, as the lure did not affect the conidial germination in the laboratory [165]. These results suggest that ICIPE 7 and ICIPE 621 could be used combined with *S. frugiperda* pheromone as an “attract and infect” strategy for sustainable management of the fall armyworm. Also, Akutse et al. [166] observed high efficacy of *M. anisopliae* isolates ICIPE 18, ICIPE 20, and ICIPE 665 against both adult and fourth instar larvae of the tomato pest *T. absoluta*, when used in combination with the *Tuta* pheromone, TUA-Optima^®^, for mass trapping and auto-dissemination. The results suggest them as promising and appropriate applicants as fungal-based biopesticides against *T. absoluta* and other solanaceous pests.

#### 7.1.2. Pheromones and Bacteria

Only a few new studies are available on pheromones and bacteria’s combined use against crop insect pests. For example, Sammani et al. [167] studied the effects of the sex pheromone components (*Z*,*E*)-9,12-tetradecadienyl acetate and (*Z*)-9-tetradecen-1-yl acetate of *Cadra cautella* (Walker) (Lepidoptera: Pyralidae) in the presence of botanical oils on insect mating, and the burrowing ability of *C. cautella* larvae in different types of flour treated with spinosad (*Saccharopolyspora spinosa*), a bacterial organism isolated from soil. The mating success was higher with botanical oils alone but declined with pheromone exposure either alone or combined with botanical oils [167]. These studies indicated that the mating and burrowing of *C. cautella* is influenced by its pheromone and by exposure to botanicals and spinosad.

For the first time, Ren et al. [168] highlighted the influence of microbial symbionts on insect pheromones and provide an example of direct bacterial production of pheromones in insects. The authors used *Bactrocera dorsalis* (Hendel) (Diptera: Tephritidae) as a model system and demonstrated that *Bacillus* sp. in the rectum of male *B. dorsalis* plays a pivotal role in sex pheromones production. They also showed that 2,3,5-trimethylpyrazine and 2,3,5,6-tetramethylpyrazine are sex pheromones produced in the male rectum.

### 7.2. Monitoring

One of the most widespread and successful applications of sex pheromones is in the detection and monitoring of pest populations [11]. Monitoring systems are based on the relationship between trap captures and the pest population or damage induced by the pest species. The number of male catches is used to establish thresholds for making decisions on when it is advisable to take treatment actions. Sex pheromones are very useful for evaluating trap catches because they are highly sensitive when detecting low insect population levels and are species-specific. These features also allow for the detection and survey of invasive species, and permit growers to perform timely insecticidal applications, thereby reducing economic and environmental costs [2].

Factors including trap design (e.g., type, color, height), trap location, type of dispenser, pheromone dose and purity, and environmental conditions during the trapping period can influence male catches by pheromone-baited traps [169,170,171,172]. In addition, it is essential to have a good knowledge of the pest biology and geographical distribution of the pest [11]. For monitoring, pheromone traps are now available for a wide range of insect pests, mostly Lepidoptera, although some are also for Coleoptera and Diptera. Some examples of using sex pheromones for monitoring Lepidoptera and Coleoptera follow.

For monitoring *Coleophora deauratella* Leinig and Zeller (Lepidoptera: Coleophoridae), an invasive pest of the red clover in Canada, field experiments were conducted to optimize several pheromone-baited trap features [169]. The type of substrate used to release the pheromone and lure age did not affect trap catches. However, moth capture in non-saturating green Unitraps was significantly higher than other traps and the authors recommended their use with either gray or red rubber septa lures.

In studies directed to monitor the presence of the banana root borer *Cosmopolites sordidus* (Germar) (Coleoptera: Curculionidae) in banana plantations of the island of Guam (USA), the male-produced aggregation pheromone (sordidin) was deployed in lure packs containing 90 mg of pheromone [170]. Ground traps were found to be superior to ramp and pitfall traps, and brown traps were more attractive to insects than other colored traps. In addition, ground traps located in the shade of the canopy were more effective than those placed in sunlight.

To optimize the monitoring of the sweetpotato weevil, *Cylas formicarius* (Fabricius) (Coleoptera: Brentidae) with the sex pheromone (Z)-3-dodecenyl-(E)-2-butenoate, several parameters affecting trap catches were evaluated [171]. Each lure contained 10 mg of pheromone and emitted the active ingredient at <0.01 mg/day. Pherocon unitraps caught higher numbers of males than ground, funnel water, and delta traps, with medium-sized traps being the most effective. The weevil preferred red over other colors, particularly light red. For optimum catches, traps should be positioned 50 cm above the crop canopy [171]. 

In monitoring studies of the pea leaf weevil, *Sitona lineatus* (L.) (Coleoptera: Curculionidae), several factors that potentially could affect capture rates of pheromone-baited traps were considered [172]. Lures contained 10 mg of pheromone and emitted the active ingredient at 0.1 mg/day. Pheromone-baited pitfall and ramp traps caught significantly more adults than ground and delta traps and pitfall traps containing gray rubber septa captured more adults than traps baited with membrane formulations.

In a dose-response field test for monitoring the processionary moth *T. pityocampa* (Denis and Schiffermüller) (Lepidoptera: Thaumetopoeidae), the number of male captures increased significantly with the dosage of the pheromone to a plateau at ca. 10 mg [173,174]. Among several trap designs, plate sticky traps showed the highest trapping efficiency and the mean trap captures correlated well with the nest density. The utilization of four plate sticky traps containing 0.2 mg of pityolure proved to be a cost-effective tool for monitoring population densities of the pest [174]. 

Recently, we have investigated the effects of air flow on the host location of adults of citrus psyllid, Diaphorina citri, and the trapping efficiency of perforated yellow trapping plate (Zeng, unpublished). It was found that the airflow influenced the movement of psyllid adults and the catching rate of the trap. The catching number with the perforated yellow plate was 45.41%, significantly higher than that of the non-perforated yellow trap plate (21.67%). These results indicated that it is important to use an air-flowable trap device baited with sex pheromones for the monitoring of certain species of insect pests.

### 7.3. Mass Trapping

Mass trapping is a direct control strategy that uses a large number of pheromone traps to reduce the population density of the target species and/or pest damage [175,176]. Compared to mating disruption, mass trapping is more efficient when both control methods have an equal number of pheromone sources [177]. This is because mating disruption only delays the searching sex, whereas traps delay them indefinitely. Mass trapping has been particularly successful in controlling large weevils in tropical crops, such as oil palms, palmito palms, plantains, and bananas. The key biological factors for success appear to be the relatively long life and slow reproductive rate of the weevils, and the fact that the aggregation pheromones attract both sexes. In the case of sex pheromones that only attract one sex, mass trapping is generally less effective despite some larger projects collecting billions of individuals [177]. Several factors, such as trap design and density, population level, biology of the target pest, isolation, and risk of immigration can influence the success of the application [10,175,178]. The pheromone composition of the target pest and the cost for its production may be also crucial for an economically feasible control by mass trapping. This is the case of the economically important click beetle *Melanotus communis* Gyllenhal (Coleoptera: Elateridae) whose female-produced pheromone is a single, readily synthesized chemical, 13-tetradecenyl acetate [179]. In addition, the cost of traps and the manual labor required to deploy them should be economically competitive to other control methods. Another factor that can affect the feasibility of mass trapping is the need for using a large quantity of pheromone that may not be cost-effective. Traps must catch 80–95% of the males to effectively reduce population [180].

Mass trapping has been mainly used against the following pests: *C. pomonella* (L.), *Zeuzera pyrina* (L.), and *Cossus cossus* (L.) in orchards; *S. littoralis* (Boisduval) and *P. gossipiella* (Saunders) in cotton and oilseed; bark beetles; palm weevils; corn rootworms; *Ephestia* spp. and *Plodia interpunctella* (Hb.) in stored products and food industries; or the gypsy moth *Lymantria dispar* (L.), and boll weevil *Anthonomus grandis* (Boheman) as invasive species [10,178,180].

Mass trapping of *T. absoluta* Meyrick (Lepidoptera: Gelechiidae), a major threat of tomato worldwide, has been implemented using homemade traps (translucent plastic cylinders) or water traps lured with a sex pheromone dispenser [181,182]. The traps are most effective when placed near ground level and, ideally, they should be loaded with 0.5 mg of pheromone. The use of 48 traps/ha reduced leaf damage more efficiently than conventional insecticide treatment.

Mass trapping of *C. sordidus* (Germar) (Coleoptera: Curculionidae) with pheromone-baited pitfall traps and *Metamasius hemipterus* (Coleoptera: Curculionidae) with pheromone-sugarcane-baited traps were conducted in commercial banana plots [183]. Capture rates of *C. sordidus* and *M. hemipterus* declined by >75 % and corn damage decreased by 61–64% in trapping plots. In addition, banana bunch weights increased 23% relative to control plots. The authors estimated that the increase in the yield value obtained was about USD $4240 per year per hectare, while the cost of the experiment was approximately USD $185 per year per hectare [183].

Control of the processionary moth by mass trapping has been pursued over the years due to the economic impact of the moth on pine forests and woodlands. In 2015, Martin [184] reported that a minimum of 4 funnel traps baited with 1 mg of the pheromone (Z)-13-hexadecen-11-ynyl acetate was necessary to be effective in a small site and 6 traps per hectare in large sites. These results were consistent with those obtained by Trematerra et al. [185] in central Italy, who reported a reduction of 53% and 79% of adults caught in 2016 and 2017 in the mass trapping parcel in comparison to the control parcel. Consequently, the average number of winter nests per tree in the trapping parcel was reduced by 88% after one year and 94% after two years, when compared to the nest reduction of 43% and 80% observed in the control parcel.

### 7.4. Mating Disruption

Mating disruption (MD) is a strategy based on the permeation of the crop with synthetic sex pheromone to disrupt chemical communication between sexes and, thus, preventing mating. To date, MD is the most developed pheromone-based technology for the direct control of moth pests [186]. The species-specificity and low toxicity of pheromone applications have led to consider MD a reliable tool for use in area-wide programs to control insect pests and manage invasive species. Microencapsulation, hand application, aerial dispensers, and matrix formulations (SPLAT, Specialized Pheromone and Lure Application Technology), have been used for pheromone emission [34]. Ideally, the dispensers should release pheromones at a constant rate, should be mechanically applicable, completely biodegradable, and made from renewable and cheap organic materials, be economically cheap, and eco-toxicologically inert [187]. The application of this technology has increased almost exponentially in the last 30 years, and it was calculated that the surface of crops being controlled for specific pests amounted to 770,000 ha in 2010 [11,188].

The most successful cases of pest control by MD are the gypsy moth *L. dispar* [186]; the codling moth *C. pomonella* [189]; the grapevine moth *Lobesia botrana* [190]; the oriental fruit moth *G. molesta* (Busck) [191]; the raisin moth *E. cautella* (Walker), the Mediterranean flour moth *Ephestia kuehniella* Zeller, and the Indian meal moth *P. interpunctella* (Hübner) [192], and the carpenter moth *Cossus insularis* (Staudinger) (Lepidoptera: Cossidae) [193].

MD has also been helpful to control plant-feeding midges that cause important crop losses in forestry and horticultural and fruit crops [2]. For instance, application of the pheromone of the swede midge *Contarinia nasturtii* Kieffer (Diptera: Cecidomyiidae) on fields of Brussels sprouts, broccoli, and cauliflower resulted in a 59–91% reduction in damage [2]. More recently, Hodgdon and coworkers [140] tested in the laboratory and in the field whether exposure to synthetic pheromone influenced female calling and the subsequent propensity to mate. In some species, females possess pheromone receptors and autodetection of their own pheromones induce them to alter their mating behavior (see Sex pheromone autodetection above). This was the case for the swede midge; while 68% of female midges mated under control conditions only 42% and 35% of females mated when pre-exposed to stereospecific and racemic three-component blends, respectively [140].

MD on tomato greenhouses has been implemented for control of *T. absoluta*. The use of 30 g/ha of pheromone can be sufficiently effective in high-containment glasshouses, with façades closed by insect-proof nets, to control the moth populations for 4 months and reduce the percentage of damaged fruits, but not in the open field or unscreened greenhouses [194,195]. The insects’ ability to undergo parthenogenesis or multiple matings stresses that immigration of mated females into greenhouses should be prevented in order to improve effectiveness of MD.

The sex pheromone of the European grape vine moth *L. botrana* in Ecoflex fibers, a cheap organic co-polyester and completely biodegradable within half a year, has been tested in MD experiments in Southwest Germany with promising results [187]. After 7 weeks of treatment, disruption effects of ca. 95% were obtained. The use of suitable mesofibers is protected by European and US patents. In this line, the authors later developed Electrospun mesofibers, novel biodegradable pheromone dispensers with diameters ranging from 0.6 to 3.5 μm. The dispensers are biodegradable and harmless to non-target organisms [196]. More recently, Luchi and coworkers [197] evaluated the efficacy of the MD products Isonet^®^ L TT and the biodegradable Isonet^®^ L TT BIO in reducing *L. botrana* damage on grapevine. Experiments were conducted in Central and Northern Italy over three years. The trials allowed a reliable control of the three generations of *L. botrana* during the whole grape-growing season.

A MD approach using pheromone puffer dispensers were considered to control *C. deauratella* at three red clover seed production fields in Alberta, Canada [198]. In all plots, aerosol-emitting pheromone puffers were able to reduce male *C. deauratella* orientation to traps by 60.7% to 93.7% compared with control plots. However, there was no corresponding decrease in larval numbers or increase in seed yield. Important challenges of this experimentation appeared to be the immigration of mated females and high population densities [198].

Univoltine species, like the processionary moth, might be ideal for MD since a single annual application might lead to population suppression without need for another application. However, the timing of application is always critical. In MD trials conducted in Aosta Valley (NW Italy) in 2016–2017, the number of males collected was significantly lower in the plots where MD was performed in comparison to control plots [199]. In addition, the total number of nests recorded per tree was significantly lower in MD plots. The technique appears to be the most appropriate control strategy for the processionary moth, and address that repeated annual applications of MD could dramatically reduce population densities below the economic injury level [199].

### 7.5. Push-Pull Strategy

The push-pull strategy, the simultaneous use of an attractant and repellent stimulus to divert pests, is an increasingly employed sustainable alternative to traditional pesticides. This strategy aims at reducing crop injury by modifying pest distribution using repellent stimuli to ‘push’ the insect pest away from the crop, and at the same time attractant stimuli to ‘pull’ the pest to other areas out of the crop. The development of push-pull strategies has been mainly directed to agricultural systems to manage insecticide resistance threats or diminish the use of insecticides. This strategy requires knowledge of insect biology, chemical ecology, and interaction between host plants and natural enemies [200]. Although there is a large variety of ‘push’ and ‘pull’ components, such as synthetic repellents, host- and non-host volatiles, host-derived semiochemicals, antifeedants, oviposition stimulants, and oviposition deterrents, among others, we will concentrate only on those strategies dealing with sex pheromones.

Sex pheromones can contribute in push-pull experiments to establish the timing of introduction of the stimuli and other population-decreasing actions [200]. Sex and aggregation pheromones attraction to herbivores can be reinforced by the synergistic action of host plant volatiles [201,202]. In push-pull trials against aphids, nepetalactone, one of the aphid sex pheromone component, and (*Z*)-jasmone a host-plant volatile that attracts aphid parasitoids, may be used to pull parasitoids into the crop [203]. In addition, the parasitoids can be pushed to the field from nearby locations by the action of tricosane and pentacosane, the lady beetle pheromone [204].

In a three years experiment in alfalfa fields, slow-release formulations of the aphid sex pheromone components (4a*S*,7*S*,7a*R*)-nepetalactone and (1*R*,4a*S*,7*S*,7a*R*)-nepetalactol significantly decreased population of the pea aphid, *Acyrthosiphon pisum* Harris [205]. At the same time, parasitism by the aphid parasitoids *Aphidius ervi* Haliday and *Praon barbatum* Mackauer was significantly increased, while no pheromone effects were detected for predators. These results show that the aphid sex pheromone can attract aphid parasitoids and enhance their ability to suppress aphid abundance in the field [205].

## 8. Conclusions

The excessive use of synthetic chemical insecticides causes pollution, pest resurgence, and resistance problems. Sex pheromones are natural insect behavior regulators that serve as suitable chemical agents in sustainable agriculture. They have the advantages over the hazardous chemicals of not killing the pest, but reducing the number of male adults, their reproduction rate, and guiding the timely application of insecticides. In the last two decades, studies have been mainly focused on the identification of new sex pheromones, characterization of sex pheromone perception mechanisms, and integration of these new advances in pheromone research to IPM programs. Mating disruption is probably the semiochemical-based technique most successfully used in IPM [137]. So far, studies on MD mechanisms have been focused on male moths almost exclusively, but studies on pheromone autodetection by females have determined that modeling MD mechanisms will increase in complexity [206]. The actual effects of the different female behaviors on MD largely remain to be understood, but their knowledge should prove useful for evaluating the potential of this strategy in pest control. From the molecular point of view, significant research is being performed on the discovery of new olfactory receptors, pheromone binding proteins, sensory binding proteins and chemical signal transduction mechanisms involved in sex pheromone communication. Advances in sequence technologies, genomics and transcriptomics of insect olfactory system will help develop new technologies for more sustainable pest management strategies, thereby reducing the use of synthetic insecticides.

## Figures and Tables

**Table 1:** Highly concerned genera on sex pheromones since 2000.

Order	Family	Genus	Publications
Lepidoptera	Noctuidae	*Helicoverpa*	194
		*Spodoptera*	173
	Tortricidae	*Grapholita*	137
		*Cydia*	128
	Plutellidae	*Plutella*	72
	Crambidae	*Ostrinia*	63
	Bombycidae	*Bombyx*	61
Diptera	Drosophilidae	*Drosophila*	49
Lepidoptera	Pyralidae	*Chilo*	42
	Tortricidae	*Lobesia*	41
	Noctuidae	*Agrotis*	40
Diptera	Tephritidae	*Bactrocera*	37
Hemiptera	Pseudococcidae	*Planococcus*	36
Lepidoptera	Lymantriidae	*Lymantria*	36
Coleoptera	Scarabaeidae	*Holotrichia*	35
Lepidoptera	Gelechiidae	*Tuta*	33
	Geometridae	*Ectropis*	30
	Lasiocampidae	*Dendrolimus*	29
Diptera	Psychodidae	*Lutzomyia*	26
Lepidoptera	Sesiidae	*Synanthedon*	25
Blattodea	Blatteidae	*Blattella*	25
Coleoptera	Buprestidae	*Agrilus*	25
Lepidoptera	Crambidae	*Pyrausta*	24
	Gracillariidae	*Phyllocnistis*	23
	Erebidae	*Hyphantria*	22
Hemiptera	Pseudococcidae	*Pseudococcus*	21
Lepidoptera	Pyralidae	*Plodia*	21
	Crambidae	*Cnaphalocrocis*	18
	Tortricidae	*Choristoneura*	18
Hemiptera	Miridae	*Apolygus*	18

**Table 2:** New sex pheromones and sex pheromone components recently identified from insect pests (2010–2020).

Pheromone/Pheromone Components	Insect	References
(*E*)-11-hexadecenal, (*E*,*E*)-10,12-hexadecadienal	*Diaphania glauculalis* (Lepidoptera: Crambidae)	Ma et al. [15]
(*E*)-10-hexadecenal, (*Z*)-10-hexadecenal, (*E*)-10-hexadecenol, (*E*,*E*)-10,12-hexadecadienal, (*Z*,*Z*,*Z*)-3,6,9-tricosatriene	*Conogethes pluto* (Lepidoptera: Crambidae)	El Sayed et al. [16]
(*Z*)-11-hexadecenyl acetate, (*Z*)-11-hexadecenal, (*Z*)-11-hexadecenol	*Trichophysetis cretacea* (Lepidoptera: Crambidae)	Pong et al. [17]
(4a*S*,7*S*,7a*R*)-nepetalactone, (1*R*,4a*S*,7*S*,7a*R*)-nepetalactol	*Hyalopterus pruni, Brachycaudus helichrysi* (Hemiptera: Aphididae)	Symmes et al. [18]
(*E*,*Z*)-3,13-octadecadienyl acetate, (*Z*,*Z*)-3,13-octadecadienyl acetate	*Synanthedon vespiformis* (Lepidoptera: Sesiidae)	Levi-Zada et al. [19]
(*E*)-11-tetradecenyl acetate, (*E*,*E*)-9,11-tetradecadienyl acetate, (*E*)-11-tetradecenol, (*E*)-11-hexadecenyl acetate	*Epiphyas postvittana* (Lepidoptera: Tortricidae)	El Sayed et al. [20]
(*Z*,*E*)-9,12-tetradecadienyl acetate, (*Z*)-9-tetradecenyl acetate, (*Z*)-11-hexadecenyl acetate, (*Z*,*E*)-9,12-tetradecadienol, (*Z*)-9-tetradecenol, (*Z*)-11-hexadecenol	*Spodoptera exigua* (Lepidoptera: Noctuidae)	Acín et al. [21]
(*Z*,*Z*)-3,13-dodecadienolide	*Parcoblatta lata* (broad wood cochroach)	Eliyahu et al. [22]
(*R*,*R*)-(*Z*)-3,7,11,15-tetramethyl hexadec-2-enal, (*R*,*R*)-(*E*)-3,7,11,15-tetramethyl hexadec-2-enal	*Dociostaurus maroccanus* (Moroccan locust)	Guerrero et al. [23]
(4,5,5)-(trimethyl-3-methylenecyclopent-1-en-1-yl)methyl acetate	*Delottococcus aberiae* (Hemiptera: Pseudococcidae)	Vacas et al. [24]
(*Z*)-9-tetradecenyl acetate, (*Z*)-9-tetradecenol, tetradecyl acetate	*Coryphodema tristis* (Lepidoptera: Cossidae)	Bouwer et al. [25]
(-)-δ-heptalactone	*Rhagoletis batava* (Diptera: Tephritidae)	Büda et al. [26]
(*E*,*E*,*Z*,*Z*)-4,6,11,13-hexadecatetraenal	*Callosamia promethea* (Lepidoptera: Saturniidae)	Gago et al. [27]
(-)-iridomyrmecin	*Leptopilina heterotoma* (Hymenoptera: Figitidae)	Weiss et al. [28]
(*Z*,*E*)-5,7-dodecadienyl acetate, (*Z*,*E*)-5,7-dodecadienol, (*Z*,*E*)-5,7-dodecadienyl propionate	*Dendrolimus tabulaeformis* (Lepidoptera: Lasiocampidae)	Kong et al. [29]
(*E*)-7,9-decadienol, (*E*)-8-decenol	*Monema flavescens* (Lepidoptera: Limacodidae)	Shibasaki et al. [30]
(1*S*,4*R*,1′*S*)-4-(1′,5′-dimethylhex-4′-enyl)-1-methylcyclohex-2-en-1-ol	*Oebalus poecilus* (Heteroptera: Pentatomidae)	Oliveira et al. [31]
(3*S*,6*S*,7*R*)-1,10-bisaboladien-3-ol, (3*R*,6*S*,7*R*)-1,10-bisaboladien-3-ol	*Tibraca limbativentris* (Hemiptera: Pentatomidae)	Blassioli-Moraes et al. [32]
